# Effect of Applying Best Practices for Physical Activity and Screen Time to Family Childcare Homes

**DOI:** 10.5888/pcd20.220325

**Published:** 2023-07-13

**Authors:** Qianxia Jiang, Patricia Markham Risica, Alison Tovar, Kristen Cooksey Stowers, Marlene B. Schwartz, Caitlin Lombardi, Sofiya Alhassan, Kim M. Gans

**Affiliations:** 1 Center for Children’s Healthy Lifestyles and Nutrition, Children’s Mercy, Kansas City, Missouri; 2 Department of Behavioral and Social Health Sciences, School of Public Health, Brown University, Providence, Rhode Island; 3 Center for Health Promotion and Health Equity, School of Public Health, Brown University, Providence, Rhode Island; 4 Department of Allied Health Sciences, University of Connecticut, Storrs, Connecticut; 5 Department of Human Development and Family Sciences, University of Connecticut, Storrs, Connecticut; 6 Rudd Center for Food Policy and Health, University of Connecticut, Hartford, Connecticut; 7 Department of Kinesiology, University of Massachusetts Amherst, Amherst, Massachusetts

## Abstract

**Introduction:**

Early childcare has been identified as an influential setting for children’s physical activity. Our objective was to determine whether children aged 2 to 5 years had more accelerometer-measured minutes of physical activity when caregivers in their family childcare home (FCCH) adhered to best practices for physical activity and screen time.

**Methods:**

We analyzed baseline 2-day observation data collected by using the Environment and Policy Assessment and Observation measure from a cluster-randomized trial. Multilevel linear regression models assessed the association between caregivers’ meeting best practices for physical activity and screen time and children’s time spent sedentary or in moderate-to-vigorous physical activity (MVPA).

**Results:**

All FCCH caregivers (N = 120) in our study were female, and 67.5% were Hispanic. Participating children (N = 349) were 52.1% female and 57.4% Hispanic. A higher score among caregivers for physical activity best practices was associated with more MVPA (B = 0.79; 95% CI, 0.02 to 1.56; *P* = .04) for children and less sedentary time (B = −2.07; 95% CI, –3.94 to –0.19; *P* = .04). A higher caregiver score for screen time best practices was associated with less sedentary time (B = −2.07; 95% CI, –3.94 to –0.19; *P* = .04) and more MVPA time (B = 0.65; 95% CI, 0.03 to .27; *P* = .04). Children in homes where caregivers offered them 60 minutes or more of outdoor play and participated in outdoor physical activity had more MVPA and less sedentary time. We found no association between various screen-time best practices and children’s sedentary time.

**Discussion:**

Children with caregivers who used more best practices for physical activity and screen time had higher activity levels and spent less time sedentary. These findings could help policy makers and people caring for young children modify existing policies and develop programs to help early childhood caregivers implement best practices to promote children’s physical activity.

**Summary**

**What is already known on this topic?**

Many children in Family Childcare Homes (FCCHs) are primarily sedentary. Caregiver practices can affect children’s activity levels; however, the connection between the home’s environment and children’s physical activity levels remain unclear.

**What is added by this report?**

We examined associations between caregiver practices and children’s physical activity in FCCHs with predominantly Hispanic providers. We found that children engaged in more physical activity in homes where caregivers met best practices related to physical activity and screen time.

**What are the implications for public health practice**

Our findings could help people involved in childcare, including policy makers, modify existing written policies and develop programs to help providers of early childcare implement best practices to promote physical activity.

## Introduction

Regular physical activity has many mental and physical health benefits for children (1). Early childhood, when children tend to be most active, is an important time for developing physical activity habits (2). Approximately 60% of preschool-aged children in the US spend some time in childcare settings (3). Early childcare settings can contribute to the development of physical activity habits by providing scheduled time for active play and creating supportive physical activity environments (1). However, most studies examining physical activity in childcare settings have been conducted in childcare centers and not in family childcare homes (FCCHs), which provide care for more than 1.6 million US children in 2005–2006 (4). In FCCHs, a professional caregiver cares for an average of 6 children in her or his home (5). Many childcare providers care for children from low-income and ethnic minority families (6).

According to evidence-based guidelines from the Nutrition and Physical Activity Self-Assessment for Child Care (NAPSACC) program and the American Academy of Pediatrics, preschoolers should engage in 60 minutes to several hours per day of unstructured physical activity and have no more than 30 minutes of screen time per week in childcare (7,8). However, many children in FCCHs do not get enough opportunities for physical activity and are primarily sedentary (9,10). Although childcare providers implement some positive physical activity and screen time practices, many of them do not fully meet best practice guidelines (11,12). Caregiver practices can affect children’s physical activity and sedentary behavior (13,14); however, links between the physical activity environment in FCCHs and children’s physical activity levels are unclear (10,15).

Furthermore, no studies have examined the associations between caregiver practices and children’s objectively measured physical activity and screen time levels in FCCHs with predominantly Hispanic caregivers. Such caregivers often care for Hispanic children, who are at higher risk for obesity than children of other ethnic groups (16,17). As our previous research showed, Hispanic caregivers may have different physical activity–related attitudes, barriers, and behaviors than non-Hispanic caregivers (12,18,19). Therefore, our study assessed whether children with predominately Hispanic childcare providers spent more time in moderate-to-vigorous physical activity (MVPA) and less sedentary time when those caregivers met evidence-based, best-practice guidelines (8). We hypothesized that if caregivers met more best practices, children would engage in more physical activity and be less sedentary.

## Methods

### Participants and family childcare homes

Our study used baseline data from the Healthy Start/Comienzos Sanos study, an 8-month cluster-randomized controlled trial designed to evaluate the efficacy of a multicomponent nutrition and physical activity intervention directed at English- and Spanish-speaking FCCHs (20). Study details are provided in Risica et al (20). The institutional review boards of Brown University and the University of Connecticut approved all study procedures and materials.

We recruited FCCHs from November 2015 through July 2018. Eligible homes had to be within 60 miles of Providence, Rhode Island, and in operation for at least 6 months. Caregivers had to be able to read and speak Spanish or English, provide meals and snacks for children, and care for at least two 2- to 5-year-old children for at least 10 hours per week. Caregivers in eligible homes completed a baseline telephone survey and an in-person survey at their home. Once we received consent from at least 1 parent of a 2- to 5-year-old child in an eligible home, a 2-day observation and measurement session was scheduled. All data collection efforts were administered by trained project staff. Caregivers received $25 for completing the baseline in-person survey and $50 for the 2-day observation. Participating children received a reusable water bottle, and parents received a $20 gift card.

### Measures

**FCCH observations.** After completion of both the telephone and in-person surveys, we conducted 2 days of observation in the FCCH by using the validated Environment and Policy Assessment and Observation (EPAO) tool (21–23), which was developed and validated to assess the practices, environments, and policies that influence children’s nutrition, physical activity, sedentary behavior, and screen time in childcare centers and FCCHs (21–23). Physical activity and screen time measures in our analysis included active and sedentary play opportunities inside and outdoors, caregiver behaviors that are supportive or unsupportive of physical activity and screen time, and education the caregiver provides to children and parents about physical activity and screen time. The observer recorded detailed notes by using an extensive, structured tool (21–23) that assessed the home environment and the caregiver’s behaviors during the visit. Extensive quality control and retraining were conducted on an ongoing basis (23).

**Self-reported physical activity and screen time in the FCCH.** We asked caregivers in the telephone survey about the frequency of physical activity or screen time, because certain related best practices (7,8) were beyond the 2-day observation window. This survey item included 4 questions from the validated NAPSACC tool (24): 1) how often (per day, week, or month) the caregiver led a planned physical activity education lesson, 2) how often the caregiver provided families with information on children’s physical activity, 3) the amount of screen time offered, and 4) how often the caregiver allowed children screen time (eg, television, computer, tablet). All responses were converted into weekly frequencies.

**Meeting best practice guidelines.** To determine whether caregivers met guidelines, we developed algorithms to compare each observed physical activity and screen time practice with its associated best practice from NAPSACC (7) or the American Academy of Pediatrics National Resource Center for Health and Safety in Child Care and Early Education and National Health and Safety Performance Standards (8). EPAO algorithms identified whether caregivers met (yes/no) the specific guidelines for total time spent in physical activity, outdoor play, adult-led physical activity, informal physical activity talks with children, sedentary time, screen time during meals, participating in indoor or outdoor physical activity with children, modeling sedentary behavior, and encouraging and prompting physical activity (Table 1). However, a few physical activity and screen time practices referred to a time frame that went beyond the 2-day observation period; thus, self-reported survey data were used in the algorithms for leading a planned physical activity education lesson at least once per week and providing families with information on children’s physical activity and screen time. For assessing the FCCH’s limiting screen time to less than 30 minutes per week, we used a combination of self-report and observational data in the algorithm. We calculated the number of physical activity (0–9) and screen time (0–5) practices met by each caregiver.

**Table 1:** Best Practices Related to Physical Activity and Screen Time Assessed Among Caregivers (N = 120) in Family Childcare Homes, Providence, Rhode Island, November 2015–July 2018^a^

Variable	Best practice	Requirement to meet best practice
Physical activity	Provide children with ≥90 minutes of daily physical activity (7,8).	Staff observer indicates that children engage in at least 90 min each day of physical activity at a level equal to or greater than easy walking.^b^
Outdoor play	Provide children with ≥60 min of daily outdoor play (7,8).	Staff observer indicates that children spend at least 60 minutes outside each day.^b^
Adult-led physical activity	Provide children with ≥45 minutes of adult-led physical activity each day (7).	Staff observer indicates that children engage in at least 45 min each day of adult-led physical activity.^b^
Physical activity education	Lead ≥1 planned physical activity lessons weekly (7).	Caregiver reports leading a planned physical activity education lesson at least once per week.^c^
Physical activity informal talk	Talk with children informally about physical activity (7).	Staff observer indicates that caregiver talks with children informally every day about the importance of physical activity a little, sometimes, or a lot.^b^
Sedentary time	Limit time children are asked to remain seated to <15 minutes daily (7).	Staff observer indicates that children were not asked to remain seated for more than 15 minutes at a time (excluding indoor play time, circle time, nap times, and TV time).^b^
Screen time	Limit screen time to <30 minutes weekly (7).	Staff observer indicates that children spent less than 30 minutes in front of a screen during the 2 observation days; caregiver reports children being allowed to spend less than 30 minutes weekly in front of screens.^b^^,^ c
Screens during meals	TV should never be on during meal or snack time (7,8).	Staff observer indicates that a TV or other screen device was not on and visible from eating area during any observed meal or snack time.^b^
Participation in indoor physical activity with kids	Always participate in indoor physical activity with children (7).	Staff observer indicates that caregiver played actively with the children a lot during indoor time on the 2 observation days.^b^
Participation in outdoor physical activity with kids	Always participate in outdoor physical activity with children (7).	Staff observer indicates that caregiver joined the children’s game outside, played with children outside, and participated in a chasing game with children a lot during outside time on the 2 observation days.^b^
Not modeling sedentary behavior	Do not model sedentary behavior (7,8).	Staff observer does not indicate that caregiver watched TV or used other screen time during the 2 observation days.^b^
Encouraging physical activity	Always prompt and praise children for being physically active (7,8).	Staff observer indicates that caregiver prompted and praised children for being physically active and prompted them to increase their physical activity a little, sometimes, or a lot during the 2 observation days.^b^
Parent communication about physical activity	Provide families with information on children’s physical activity (7,8).	Caregiver reports giving families information on 1) the amount of time children should spend being physically active, 2) encouraging children to be physically active, 3) limiting long periods of seated time for children, 4) the amount of time children should spend playing outdoors, and 5) using the outdoors to encourage children’s active play.^c^
Parent communication about screen time	Provide families with information on screen time for children (7,8).	Caregiver reports giving families information on 1) the amount of screen time children should have, 2) why it is important to limit screen time, and 3) other activities for children instead of screen time.^c^

Abbreviations: AAP, American Academy of Pediatrics; EPAO, Environment and Policy Assessment and Observation; NAPSACC, Nutrition and Physical Activity Self-Assessment for Child Care.

a Based on NAPSACC guidelines (8) and an AAP algorithm (7). Best practices were based on EPAO observational data or caregiver-reported survey data.

b From EPAO (21–23) observations.

c From telephone survey of caregivers.

**Accelerometer measurement of children’s physical activity.** Children’s physical activity at the FCCH was measured by using an accelerometer (Actilife software, Actigraph) for 2 days. Accelerometers were placed on a belt around the child’s waist by the observer at the start of each day and removed before children left to go home (9). Because of time constraints in the FCCH setting, we established minimum wear criteria (ie, ≥1 day of wear, ≥3 hours of wear during the FCCH day) (25). Mean accelerometer wear time was 5.6 hours, and median wear time was 6.3 hours (9), adequate time to capture physical activity during the child’s time in care. Five-second epochs were used to better detect short bursts of physical activity, and appropriate cut-points for this age group were used to categorize activity as sedentary, light, moderate, vigorous, or moderate to vigorous based on metabolic equivalents (2,9,25). Day-level data for each child were averaged and then standardized into minutes per hour to account for variation in the length of the FCCH day and children’s wear time. Data were scored to create variables associated with time and percentage of observed time each child spent in sedentary, light, moderate, vigorous, and moderate to vigorous activity across the 2-day observation period, not including naptime. Primary outcomes were time spent in the MVPA and sedentary categories.

### Analysis

We constructed multilevel linear regression models to assess the association between the number of physical activities and screen time practices met by caregivers and the time children spent sedentary or in MVPA. Multilevel linear regression models were then used to access the association between the caregiver meeting specific physical activity and screen time best practices and children’s MVPA and sedentary time. The resulting models were presented as parameter estimates, with 95% CIs and 2-sided *P* values. All models included adjustment for FCCH cluster and covariates that were of a priori interest based on previous evidence or theoretical associations (ie, caregiver ethnicity and income level) to help reduce confounding and the risk of including variables that could increase bias. The Bonferroni correction was used to control for multiple comparisons, and the adjusted *P* value was *P* = .004 (except for the count of physical activity and screen time practices met by caregivers). All analyses were conducted in Stata SE 16 (StataCorp LLC).

## Results

### Participants

Our sample included 120 female caregivers (67.5% Hispanic, 42.5% White, 15.0% Black, 75.0% married or living with a partner). Their average age was 48.9 years (SD = 9.0), and 13.3% had an annual household income less than $25,000. Less than half (43.3%) had a high school diploma or GED (general education development) or less, and 82.5% accepted Child and Adult Care Food Program subsidies (Table 2). Our sample included 349 children (52% girls, 57% Hispanic, 46% White or Caucasian, and 10% Black). The average age was 3.5 (SD, 0.98) years. Most ate breakfast (84%) and lunch (97%) at the FCCH. On average, children spent 7.6 (SD, 0.83) hours per day at the FCCH (Table 3).

**Table 2:** Demographic Characteristics of Caregivers (N = 120) in Family Childcare Homes, Providence, Rhode Island, November 2015–July 2018

Characteristic	Value
**Sex, n (%)**	
Female	120 (100)
**Age, mean (SD), y**	48.9 (9.0)
**Ethnicity, n (%)**	
Hispanic	81 (67.5)
Non-Hispanic	39 (32.5)
**Race, n (%)**	
White/Caucasian	51 (42.5)
Black	18 (15.0)
American Indian	4 (3.3)
Native Hawaiian	3 (2.5)
Other	28 (23.3)
Multiple races	3 (2.5)
Unknown	13 (10.8)
**Country of birth, n (%)**	
US	35 (29.2)
Non-US	85 (70.8)
**Marital status, n (%)**	
Single	11 (9.2)
Married or living with a partner	90 (75.0)
Divorced	10 (8.3)
Separated	5 (4.2)
Widowed	4 (3.3)
**Annual household income, n (%), $**	
–25,000	16 (13.8)
25,000–50,000	57 (49.1)
50,000–75,000	24 (20.7)
75,000–100,000	12 (10.3)
≥100,000	7 (6.0)
**Highest level of education, n (%)**	
Less than high school diploma	13 (10.8)
High school diploma or GED	39 (32.5)
Associate degree	46 (38.3)
Bachelor’s degree	18 (15.0)
Master’s degree or higher	4 (3.3)
**Accept CACFP subsidies, n (%)**	99 (82.5)
**Hours worked per week as a provider, mean (SD)**	62.4 (13.8)
**Number of children in care (including own children or grandchildren), mean (SD)**	7.7 (3.1)
**Years working in early childcare, mean (SD)**	12.8 (8.4)

Abbreviations: CACFP, Child and Adult Care Food Program; GED, general educational development.

**Table 3:** Demographic Characteristics of Children (N = 349) in Family Childcare Homes (FCCHs), Providence, Rhode Island, November 2015–July 2018

Category	Value
**Sex, n (%)**	
Male	167 (47.9)
Female	182 (52.1)
**Age, mean (SD), y**	3.5 (0.98)
**Ethnicity, n (%)**	
Hispanic	195 (57.4)
Non-Hispanic	145 (42.6)
**Race, n (%)**	
White/Caucasian	161 (46.3)
Black	33 (9.5)
American Indian	3 (0.9)
Native Hawaiian	3 (0.9)
Asian	3 (0.9)
Other	98 (28.2)
Multiple races	137 (31.3)
**Child eats breakfast at FCCH, n (%)**	295 (84.5)
**Child eats lunch at FCCH, n (%)**	340 (97.4)
**Child eats dinner at FCCH, n (%)**	32 (9.2)
**Hours per day at FCCH, mean (SD)**	7.6 (0.83)

### Implementation of recommended physical activity practices

Caregivers in our sample did not meet best practices for physical activity and screen time as described in our prior article (12). Caregivers implemented on average 2.1 (B = 0.97) of 9 physical activity–related best practices and 2.5 (B = 0.93) of 5 screen time best practices. Children spent on average 10.1% (B = 4.93) of their time in MVPA and were sedentary 61.5% (B = 11.54) of the time. Children spent 0.8% more time in MVPA (B = 0.79; 95% CI, 0.02 to 1.56; *P* = .04) and 2.1% less time being sedentary (B = −2.07; 95% CI, –3.94 to –0.19; *P* = .04) when cared for by a caregiver who met 1 additional physical activity or screen time-related best practice (Table 4). Children with caregivers who met the following 3 physical activity best practices had more MVPA time than children whose caregivers did not meet these guidelines: 1) providing children with 60 minutes or more of daily outdoor play (B = 2.29; 95% CI , 0.79 to 3.79; *P* = .003), 2) participating in indoor physical activity with children (B = 1.86; 95% CI, 0.74 to 2.98; *P* = .001), and 3) always participating in outdoor physical activity with children (B = 3.16; 95% CI, 1.00 to 5.31; *P* = .004). Children in FCCHs where the following 2 physical activity best practices were implemented had less sedentary time compared with children in homes that did not: 1) providing children with 60 minutes or more of daily outdoor play (B = −7.72; 95% CI, –11.35 to −4.08; *P* < .001) and 2) participating in outdoor physical activity with children (B = −8.50; 95% CI, –11.50 to –5.51; *P* < .001). 

**Table 4:** Relationship Between Caregiver (N = 120) Practices, Children’s (N = 349) Moderate-to-Vigorous Physical Activity (MVPA), and Sedentary Time, Providence, Rhode Island, November 2015–July 2018^a^

Caregiver practice^b^	Change in children’s behavior	
	MVPA time, B (95% CI) [*P *value]	Sedentary time, B (95% CI) [*P *value]
**Physical activity–related practices**		
No. of physical activity practices met by providers	0.79 (0.02 to 1.56) [.04]	−2.07 (–3.94 to –0.19) [.04]
Provided children with 90 min or more of indoor or outdoor physical activity each day	.002 (–1.36 to 1.36) [.99]	0.45 (–3.66 to 4.57) [.83]
Provided children with 60 min or more of outdoor play each day	2.29 (0.79 to 3.79) [.003]	−7.72 (–11.35 to −4.08) [<.001]
Provided children with 45 min or more of adult-led physical activity each day	2.75 (–1.39 to 3.05) [.19]	−8.25 (–20.29 to 3.78) [.18]
Led a planned physical activity class one or more times per week	−1.91 (–0.64 to 2.69) [.05]	3.48 (–0.54 to 7.50) [.09]
Talked with children informally about the importance of physical activity	1.16 (–0.76 to 3.07) [.23]	−1.83 (–7.42 to 3.76) [.52]
Always participated in indoor physical activity with children	1.86 (0.74 to 2.98) [.001]	−2.34 (–7.61 to 2.92) [.38]
Always participated in outdoor physical activity with children	3.16 (1.00 to 5.31) [.004]	−8.50 (–11.50 to −5.51) [<.001]
Always prompted and praised children for being physically active	1.99 (–0.02 to 4.01) [.05]	3.06 (–2.14 to 8.26) [.25]
Provided families with information on children’s physical activity	0.30 (–1.08 to 1.69) [.67]	−1.14 (–4.66 to 2.38) [.52]
**Screen time–related practices**		
Count of screen time practices met by providers	0.65 (0.03 to 1.27) [.04]	−2.07 (–3.94 to –0.19) [.04]
Limited the time children are asked to remain seated to less than 15 min a day	1.42 (–0.79 to 3.62) [.21]	−4.86 (–10.72 to 1.00) [.10]
Limited children’s screen time to less than 30 min per week	0.47 (–0.94 to 1.88) [.51]	−1.47 (–5.31 to 2.37) [.45]
TV was never on during meal or snack	0.90 (–0.62 to 2.42) [.24]	−2.36 (–6.14 to 1.43) [.22]
Did not model sedentary behavior	0.52 (–0.85 to 1.88) [.46]	−1.35 (–4.99 to 2.30) [.47]
Provided families with information on children’s screen time	–0.15 (–1.50 to 1.20) [.82]	0.06 (–3.40 to 3.52) [.97]

a All models were controlled for provider ethnicity, income, and Bonferroni correction (except for the number of physical activity or screen time practices met by caregivers). The adjusted critical value was *P* = .004.

b Reference group is not meeting specific practices (except for the number of physical activity or screen time practices met by caregivers).

### Caregivers’ implementation of recommended screen time practices

Children in FCCHs with caregivers who implemented more screen time–related best practices experienced less sedentary time (B = −2.07; 95% CI, –3.94 to –0.19; *P* = .04) and more MVPA time (B = 0.65; 95% CI, 0.03–1.27; *P* = .04) in adjusted multiple linear models. However, we found no significant associations between children’s sedentary time or MVPA time and implementing specific screen-time best practices (Table 4).

## Discussion

In our study, family caregivers on average implemented only 2 of 9 physical activity–related best practices. For every physical activity practice met by caregivers, the percentage of children’s MVPA time increased by 0.8%, and the percentage of their sedentary time decreased by 2.1%. Physical activity during the preschool years is associated with many health benefits (26). Even replacing 10 minutes of time (2% of the childcare day) spent sitting with MVPA is related to better health in children (27). Furthermore, children’s physical activity levels during their early years can track into adolescence and adulthood where they are strongly related to risk of chronic diseases (26–28). In adulthood, increasing MVPA by even as little as 5 to 10 minutes per day can have positive health benefits (29–31). Given that young children spend most of their waking hours in childcare, intervening with caregivers, including those in FCCHs, to promote physical activity and its associated health benefits is strongly recommended (26).

Our previous research found that the following physical activity best practices were least likely to be met by caregivers (ie, met by less than 20% of caregivers): providing children with at least 60 minutes of outdoor play daily, providing children with at least 45 minutes of adult-led physical activity each day, participating in outdoor physical activity with children, participating in indoor physical activity with children, prompting and praising children for being active, and talking with children informally about the importance of physical activity (12).

Our analysis found that meeting best practices related to providing enough outdoor physical activity time and the caregiver leading both indoor and outdoor play were significantly associated with more MVPA levels and less sedentary time. This finding agrees with previous studies that reported that frequency of outdoor play and opportunities for structured physical activity were strongly associated with higher physical activity levels in both center-based childcare settings and FCCHs (13,32,33). Generally, outdoor play has been shown to be a strong predictor of MVPA among young children (14,34). In addition, we found that children were more active in FCCHs when caregivers participated in outdoor physical activity with them. This finding is consistent with an Oregon study that showed that children were more active when caregivers regularly engaged in active play with them (33). However, some studies conducted in center-based childcare settings reported that caregiver behavior during active play sessions was not significantly related to children’s MVPA levels (32,35,36). These findings suggest that caregivers engaging in best physical activity practices might be more influential in FCCHs because of the smaller number of children and lack of play space and equipment (33). Thus, more research should focus on how to encourage caregivers to participate with or lead children in outdoor play.

We also found in our study that children were more active in FCCHs if caregivers participated in indoor physical activity with them. Caregivers’ encouragement of indoor play in childcare centers has been associated with children’s physical activity (14). Similarly, some previous studies of FCCHs reported that caregivers’ sufficient use of indoor play space to engage in active play was significantly associated with children having more MVPA (15,33) and less sedentary time (13). Therefore, encouraging caregivers to participate in children’s indoor physical activity and to interact with them may help promote active lifestyles in children. However, lack of indoor play space has been consistently identified as a barrier to promoting physical activity (15,33). Providing more resources and training opportunities related to the effective use of indoor space and play equipment may be helpful to FCCH caregivers in promoting children’s indoor physical activity.

In our study, for every screen time practice met by caregivers, the percentage of child MVPA time increased by 0.7%, and the percentage of child sedentary time decreased by 2.1%. Our previous research found that although more than half of caregivers in FCCHs met guidelines for no television viewing during the child’s meal and snack time and providing families with information about children’s screen time, less than a third of caregivers limited screen time to less than 30 minutes per week (12). In another study, less sedentary time was found among children attending FCCHs that met best practices related to reducing children’s sitting time and screen time (13). In addition, a study conducted in center-based childcare settings found that policies and practices limiting screen time in childcare centers were significantly related to the reduction of children’s sedentary time (35). Therefore, caregivers in FCCHs should be encouraged to limit screen time and provide a supportive indoor and outdoor play environment to further reduce sedentary time among children.

We found that the most important caregiver practice in promoting children’s physical activity was leading the physical activity and active play indoors and outdoors. Future interventions and training should support caregivers in implementing these physical activity–related best practices. However, such interventions should also acknowledge the barriers that caregivers may face related to implementing these practices. For example, lack of indoor play space has been consistently identified as a barrier to promotion of physical activity in FCCHs (18). Compared with center-based early childcare settings, FCCHs usually include children at different developmental stages. Furthermore, caregivers’ low self-efficacy to conduct physical activity themselves may influence their ability to model it for children (12). Thus, providing resources and training to caregivers in FCCHs in how to effectively use indoor space and play equipment can promote children’s indoor physical activity.

Outdoor play in general and neighborhood-level inequities (eg, access to parks and playgrounds) are also clearly linked (37,38). Thus, equity issues need to be considered. Our study had a high proportion of Hispanic caregivers, and Hispanic populations are more likely to live in neighborhoods that lack access to parks and playgrounds than are White populations (39). Furthermore, our previous research among this sample found that Hispanic caregivers were more likely to perceive barriers related to children’s safety while playing outside (18). A previous focus group study with Hispanic caregivers also found concerns about safety and injuries related to exercising indoors in small spaces as well as concerns related to being outside in cold or rainy weather (19). Hispanic caregivers also felt it was difficult to get children of different ages involved in group physical activity (19). Therefore, policy makers need to consider both environmental and cultural barriers that may prevent caregivers from implementing physical activity best practices.

Several opportunities exist to use policy to improve physical activity environments in FCCHs, for example, the guidelines for physical activity and screen time of the federal Child and Adult Care Food Program (22). Though some states have guidelines for physical activity and screen time (40), not all do. States may use quality rating systems to help early childcare providers meet higher standards. For example, Rhode Island has the BrightStars (https://brightstars.org) rating system to help families access quality childcare. Such systems could encourage caregivers to implement physical activity best practices by including these practices in their rating criteria (41). States also require continuing education for childcare providers, which could be used to train them in the best practices identified in our study. Implementing evidence-based guidance in FCCHs could meaningfully improve the opportunity to develop healthy activity patterns for millions of US children.

Our study had several limitations. We purposely over-recruited Hispanic caregivers because they have been ignored in most prior research. Thus, selection bias may be a concern, and results may not be representative of all FCCHs. Because only baseline data were analyzed, we cannot provide cause-and-effect interpretations of the data. Although the use of accelerometers to assess physical activity and our observation methods for assessing physical activity and screen time practices are both study strengths, we had only 2 days of data; thus, these data may not be completely representative of caregivers’ usual physical activity and screen time practices and children’s usual physical activity patterns in the childcare setting. Furthermore, our binary scores likely did not capture the variability of activity-related practices in FCCHs, which would decrease the likelihood of finding significant differences in child activity levels.

### Conclusion

Childcare providers play a crucial role in caring for young children and influencing their physical activity (1,26). Our findings suggest an opportunity for more training of caregivers in FCCHs related to physical activity and screen time. Caregivers’ participation in both indoor and outdoor play with children can increase activity levels among children. Our results highlight the need for interventions to support caregivers in implementing practices related to providing sufficient indoor and outdoor play, effectively using indoor play space, and limiting screen time. Our findings could also inform efforts of interested parties and policy makers to modify existing written policies and develop programs to further help implement best practices that promote physical activity in early childcare settings.
